# Aqueous Extract of *Agaricus blazei* Murrill Prevents Age-Related Changes in the Myenteric Plexus of the Jejunum in Rats

**DOI:** 10.1155/2015/287153

**Published:** 2015-04-16

**Authors:** Ana Paula de Santi-Rampazzo, João Paulo Ferreira Schoffen, Carla Possani Cirilo, Mariana Cristina Vicente Umada Zapater, Fernando Augusto Vicentini, Andréia Assunção Soares, Rosane Marina Peralta, Adelar Bracht, Nilza Cristina Buttow, Maria Raquel Marçal Natali

**Affiliations:** ^1^Department of Morphological Sciences, State University of Maringá (UEM), 87020-900 Maringá, PR, Brazil; ^2^Center of Biological Sciences, State University of the North of Paraná (UENP), Bandeirantes, PR, Brazil; ^3^Department of Biochemistry, State University of Maringá (UEM), 87020-900 Maringá, PR, Brazil

## Abstract

This study evaluated the effects of the supplementation with aqueous extract of *Agaricus blazei* Murrill (ABM) on biometric and blood parameters and quantitative morphology of the myenteric plexus and jejunal wall in aging Wistar rats. The animals were euthanized at 7 (C7), 12 (C12 and CA12), and 23 months of age (C23 and CA23). The CA12 and CA23 groups received a daily dose of ABM extract (26 mg/animal) via gavage, beginning at 7 months of age. A reduction in food intake was observed with aging, with increases in the Lee index, retroperitoneal fat, intestinal length, and levels of total cholesterol and total proteins. Aging led to a reduction of the total wall thickness, mucosa tunic, villus height, crypt depth, and number of goblet cells. In the myenteric plexus, aging quantitatively decreased the population of HuC/D^+^ neuronal and S100^+^ glial cells, with maintenance of the nNOS^+^ nitrergic subpopulation and increase in the cell body area of these populations. Supplementation with the ABM extract preserved the myenteric plexus in old animals, in which no differences were detected in the density and cell body profile of neurons and glial cells in the CA12 and CA23 groups, compared with C7 group. The supplementation with the aqueous extract of ABM efficiently maintained myenteric plexus homeostasis, which positively influenced the physiology and prevented the death of the neurons and glial cells.

## 1. Introduction

Aging is associated with a progressive decline in physiological function and metabolic processes [1]. The causes of this decline are linked to immune system dysfunction and disorders of energy metabolism that create oxidative stress [2]. Oxidative stress occurs in cell systems whenever the production of free radical molecules exceeds antioxidant capacity. If not removed, free radicals attack and damage proteins, lipids, and nucleic acids, diminishing their activity and leading to losses in energy metabolism, cell signaling, transport, and other important functions [3], in addition to their role in cellular death through necrosis or apoptosis [4]. To minimize the impact of an imbalance between reactive oxygen species and antioxidants, investigations of substances with possible antioxidant capacity have garnered significant scientific interest.

The benefits of ingesting traditional mushrooms are widely recognized, and these mushrooms are used worldwide as food supplements. To evaluate their effects, studies have been performed using basidiomycetes, particularly the species* Agaricus blazei *Murrill (ABM; known popularly as* cogumelo do sol*). These studies have focused on both nutritional and pharmacological objectives and assessed possible antioxidant properties and the prevention of various diseases, including cancer, diabetes, hyperlipidemia, arteriosclerosis, and chronic types of hepatitis [5].

Variations occur in the gastrointestinal tract during development and senescence. These changes involve structural and functional changes, such as decreases in the frequency and amplitude of peristaltic movements, digestion, nutrient absorption, and cell immunity [6, 7]. Although some studies have suggested maintenance of the structure of the intestinal tunica during aging [8], other reports have indicated that aging can alter villus height, crypt depth, and muscle layer thickness [9, 10].

Functional impairment of the gastrointestinal tract is directly linked to changes in extrinsic nerve components (i.e., sympathetic and parasympathetic peripheral nerve fibers) and an intrinsic component (i.e., the enteric nervous system [ENS]). This system modulates complex functions, such as motility, secretion, and blood flow. It consists of a ganglionated plexus and two ganglionated plexi: the myenteric plexus (with ganglia located between the layers of smooth muscle of the muscular tunica) and the submucosal plexus (which has its ganglia in the submucosal tunica) [11].

Aging causes a reduction of the number of neurons in the ENS. This loss is associated with not only an increase in free radicals [12] but also a reduction of neurotrophic factors that originate in glial cells, which are essential for neuronal development and maintenance [13]. Moreover, previous studies have found significant age-related increases in cell body area [8, 14], which may be justified by rearrangement of the remaining neurons, demonstrating the neuroplasticity of fully differentiated tissue.

Considering the possible antioxidant potential of edible and medicinal mushrooms, the objective of the present study was to evaluate the effects of an aqueous extract of* Agaricus blazei* on the morphology and intrinsic innervation of the intestine and myenteric plexus in aging rats.

## 2. Materials and Methods

### 2.1. Obtaining* Agaricus Blazei* Murrill (ABM) Extract

The present study used dehydrated basidiomes of ABM produced in Ibema, PR, Brazil (25°6′50′′ south, 53°0′53′′ west). The basidiomes were milled to form a fine powder and then subjected to aqueous extraction, modified from the methodology of Soares et al. [15]. Distilled water (100 mL) was added to every 10 g of milled basidiome, which remained under agitation at 28°C for 3 h. The residual solids were removed by vacuum filtration using size-1 Whatman filter paper and again subjected to extraction, which was repeated three times. The filtrates were lyophilized and kept in a freezer at −20°C. The chemical characterization of aqueous extract of the* Agaricus blazei* is described by Soares [16].

### 2.2. Animals

Starting at 7 months of age, 25 male Wistar rats (*Rattus norvegicus*) were housed in polypropylene boxes (four individuals per box) in the Animal House in the Morphological Sciences Department under a 12 h/12 h light/dark cycle at 22 ± 2°C. The rats were assigned to five groups: 7 months of age (C7), 12 months of age (C12 and CA12), and 23 months of age (C23 and CA23). All of the procedures in this study that involved the use of animals were approved by the Committee for Ethics in Animal Experimentation of Maringá State University (procedure number 063/2010).

### 2.3. Treatment and Euthanasia

The animals were fed* ad libitum* with standard rodent chow (NUVILAB, NUVITAL). The rats in the CA12 and CA23 groups were supplemented daily via gavage with 1 mL of an aqueous solution that contained 26 mg of freeze-dried ABM beginning at 7 months of age.

The study evaluated body weight, food intake by offering 100 g daily per animal and calculating the remainder, and water intake by offering 300 mL per day and calculating the remainder throughout the experimental period. At 7, 12, and 23 months of age, the animals intravenously received vincristine sulfate (0.5 mg/kg body weight), a protein synthesis blocker, 2 h prior to euthanasia. The injections occurred at the same time each day (6:00 AM) in all of the experiments to avoid circadian variations.

Afterwards, the animals were intraperitoneally anesthetized with sodium thiopental (Thionembutal, Abbott Laboratories, North Chicago, IL, USA) at a dose of 40 mg/kg of body weight, and the nasoanal length was measured to determine the Lee index (body weight^1/3^ (g)/nasoanal length (cm) × 1000). Following blood collection by cardiac puncture for biochemical analyses, the animals were euthanized by an overdose of anesthetic. Laparotomy was performed to remove and measure the length of the small intestine and weigh periepididymal and retroperitoneal adipose tissues. Jejunum samples were sent for histological processing and immunohistochemical techniques to study the myenteric plexus.

### 2.4. Biochemical Analysis of Blood Components

For the analysis of total proteins, albumin, globulins, triglycerides, and total cholesterol, blood was collected and placed in a test tube to obtain serum. To measure glucose levels, the blood was kept in a test tube that contained ethylenediaminetetraacetic acid (EDTA) fluorinated at a rate of 50 *μ*L/3 mL to obtain plasma and nonfluorinated EDTA to analyze aspartate aminotransferase (AST) and alanine aminotransferase (ALT) enzymes and total plasma antioxidant capacity (TAC-ABTS [2,2′-azino-di(3-ethylbenzthiazoline-6-sulfonic acid)]). The samples were centrifuged at 3000 rotations per minute for 15 min, and the levels were determined using Analisa kits (Gold Analisa Diagnóstica Ltda, Minas Gerais, Brazil). TAC-ABTS was assessed according to the methodology described by Erel [17].

### 2.5. Tissue Processing and Histological Analysis

Samples of the jejunum were opened at the mesenteric border, fixed in Bouin's solution (750 mL saturated picric acid solution, 250 mL formaldehyde, and 50 mL glacial acetic acid) for 6 h, stored in 70% alcohol, and subjected to the following procedures.

#### 2.5.1. Paraffin Inclusion

The jejunum samples were dehydrated in a series of increasing alcohol concentrations, cleared in xylol, and embedded in paraffin to obtain 7 *μ*m-thick semiserial histological sections using a Leica RM 2145 microtome. These sections were then stained with hematoxylin-eosin (H&E) to evaluate the thickness of the mucosa tunic, muscular coat, and total intestinal wall.

Morphometric analyses were performed by sampling images captured using a 10x lens on an Olympus BX41 optical microscope coupled to a high-resolution Olympus Q Color 3 camera. Thicknesses were estimated by measuring 10 random points per section, for a total of 100 measurements per animal, using ImagePro Plus 4.5 image analysis software (Media Cybernetics). The results are expressed as micrometers.

#### 2.5.2. Historesin Inclusion

The jejunum samples were dehydrated in 95% alcohol, 100% alcohol, and 100% alcohol + infiltration solution (resin activator) at a ratio of 1 : 1 and stored overnight at −4°C in infiltration solution. The samples were then placed inside specific containers with solution for inclusion (infiltration solution + hardener) and oven-dried at 37°C for approximately 10 days. The blocks were then subjected to microtomy to obtain 2.5 *μ*m-thick semiserial sections, stained with H&E to morphometrically analyze the villi, intestinal crypts, and metaphase index (MetI), and then subjected to the periodic acid-Schiff (PAS) histochemical technique to identify globet cells.

*(1) Measuring Villi and Intestinal Crypts*. The heights of 90 villi and 90 crypts per animal were measured longitudinally from images of the mucosa captured using a 10x lens on an Olympus BX41 optical microscope coupled to a high-resolution Olympus Q Color 3 camera with ImagePro Plus 4.5 image analysis software (Media Cybernetics). The results are expressed as micrometers.

*(2) Metaphase Index*. The MetI is expressed as the percentage of metaphase nuclei divided by the total number of counted nuclei obtained in longitudinal crypts with visible lumen. A total of 2,500 cells per animal were quantified using an Olympus BX41 light microscope (Tokyo, Japan) with a 40x lens. The MetI was multiplied by Tannok's constant (Kt = 0.57) to correct tissue geometry and avoid overestimating the number of metaphases [18]. The following equation was used to calculate the MetI: MetI = number of cells in metaphase × 100 × Kt/total number of cells in crypts.

*(3) Histochemical Analysis of Globet Cells*. The semiserial sections were subjected to the PAS histochemical technique to quantify the population of goblet cells in 50 microscopic fields (0.352 mm^2^/field) per animal. The quantitative analyses were performed with images obtained with an Olympus BX41 optical microscope coupled to a high-resolution Olympus Q Color 3 camera. The cells were counted with the aid of ImagePro Plus 4.5 software (Media Cybernetics).

### 2.6. Morphoquantitative Analysis of the Myenteric Plexus

#### 2.6.1. Obtaining Membrane Preparations

Jejunum samples were washed with 0.1 M phosphate-buffered saline (PBS; pH 7.4) to remove any residue, filled with Zamboni's fixative, tied at the end, submerged in the same fixative, and kept in cool storage for 18 h. They were then opened and subjected to dehydration using increasing concentrations of alcohol (95% and 100%), cleared in xylol, and rehydrated in a decreasing series of alcohol concentrations (100%, 90%, 80%, and 50%). They were then stored in 0.1 M PBS (pH 7.4) with 0.08% sodium azide at 4°C. Total preparations of the muscle tunica were obtained by microdissection of the samples with a stereoscope with transillumination to remove the mucosa and submucosa tunics.

#### 2.6.2. Double HuC/D-nNOS and HuC/D-S100 Immunolabeling

The total preparations of the jejunum muscle tunica were subjected to immunohistochemical techniques to detect HuC/D protein [19], neuronal nitric oxide synthase (nNOS) enzyme [20], and S100 protein [21]. The membranes were rinsed twice in 0.1 M PBS (pH 7.4) with 0.05% Triton X-100 for 10 min and immersed for 1 h in a solution that contained 0.1 M PBS (pH 7.4), 0.05% Triton X-100, 2% bovine serum albumin (BSA), and 10% goat serum to avoid nonspecific binding. The tissues were then incubated for 48 h in a solution that contained 0.1 M PBS (pH 7.4) with 0.05% Triton X-100, 2% BSA, 2% goat serum, and primary antibodies (Table 1). The membranes were washed three times in 0.1 M PBS (pH 7.4) with 0.05% Triton X-100 for 5 min, and incubated for 2 h at room temperature with secondary antibodies (Table 1). The preparations were washed with 0.1 M PBS (pH 7.4) and arranged between the slides with ultrapure glycerol.

#### 2.6.3. Morphoquantitative Analysis

To quantify the HuC/D^+^ myenteric neuron population, nNOS^+^ subpopulation, and S-100^+^ glial cells, all of which are immunofluorescent, an Olympus BX40 light microscope was used, fitted with specific immunofluorescence filters and coupled to a Moticam 2500 camera. The density (expressed as cells/cm^2^) was calculated by counting neuronal and glial cell bodies in microscopic images (32 images/animal) captured from the middle region (60°–120°; 240°–300°) of the intestinal circumference, considering 0° as the mesenteric insertion [22], using a 20x lens. The area of each analyzed image was 0.093 mm^2^. Neurons and glial cells were also counted in 50 ganglia per animal in the C7, C23, and CA23 groups. For the morphometric analysis, cell body areas (*μ*m^2^) were measured in 100 HuC/D neurons^+^ and 100 S-100^+^ glial cells per animal and 70 nNOS^+^ cell bodies per animal using ImagePro Plus 4.5 software (Media Cybernetics).

### 2.7. Statistical Analysis

The data were analyzed for normality using the Kolmogorov-Smirnov test. The parametric data were subjected to one-way analysis of variance (ANOVA) followed by Tukey's* post hoc* test using Prism 5.0 software (GraphPad, San Diego, CA, USA). The nonparametric data were analyzed using a block design with Statistica software (StatSoft) followed by Tukey's* post hoc* test. The significance level was 5%, and the results are expressed as mean ± standard error.

## 3. Results

### 3.1. Biometric Parameters

Body weight, food and water intake, the Lee index, periepididymal and retroperitoneal fat weight, and small intestine length are presented in Table 2.

### 3.2. Biochemical Analysis of Blood Components

**Figure 1:**
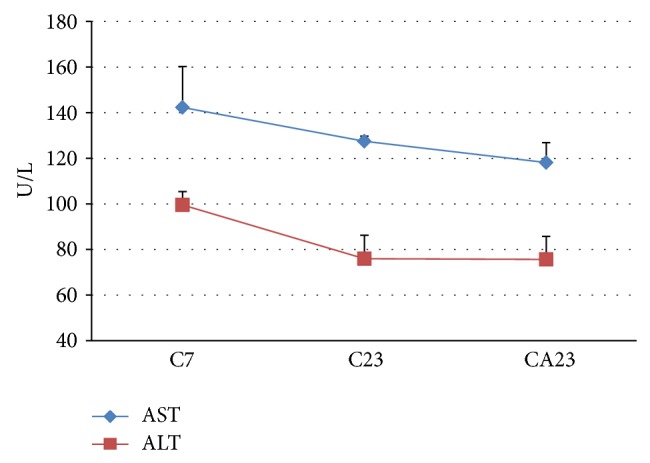
Plasma levels of the enzymes aspartate aminotransferase (AST) and alanine aminotransferase (ALT) in rats at 7 months of age (C7 group) and 23 months of age (C23 group) and 23-month-old rats supplemented with the aqueous extract of* A. blazei *(CA23 group). The results are expressed as mean ± standard error.

**Figure 2:**
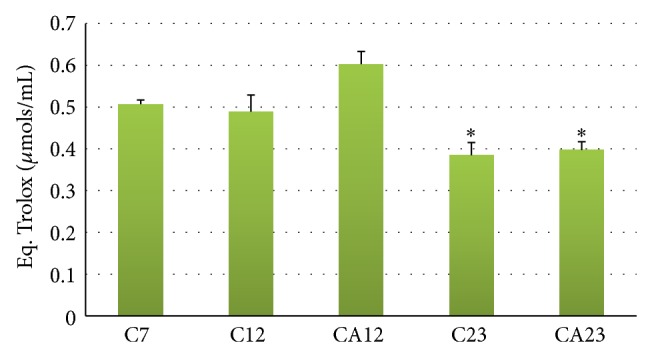
Total plasma antioxidant capacity (TAC-ABTS) in aging rats (C7, C12, and C23 groups) and aging rats supplemented with the aqueous extract of* A. blazei *(CA12 and CA23 groups). ^*^ *P* < 0.05, compared with C7 group. The results are expressed as mean ± standard error.

The blood levels of total cholesterol, total proteins, globulins, albumin, triglycerides, and glycemia are shown in Table 3. The plasma levels of the liver enzymes AST and ALT, which were evaluated at the beginning (C7) and end (C23 and CA23) of the experiment, were not significantly different between groups (Figure 1). The total antioxidant capacity of plasma (TAC-ABTS) was significantly reduced in 23-month-old animals. ABM supplementation did not significantly differ between the C23 and CA23 groups. A tendency (*P* > 0.05) toward an improvement in antioxidant capacity was observed in supplemented 12-month-old animals, but the difference between the C7 and C12 groups was not significant (Figure 2).

### 3.3. Histological Analysis

The intestinal morphometry results, MetI, and number of goblet cells are shown in Table 4.

### 3.4. Morphoquantitative Analysis of the Myenteric Plexus

**Figure 3:**
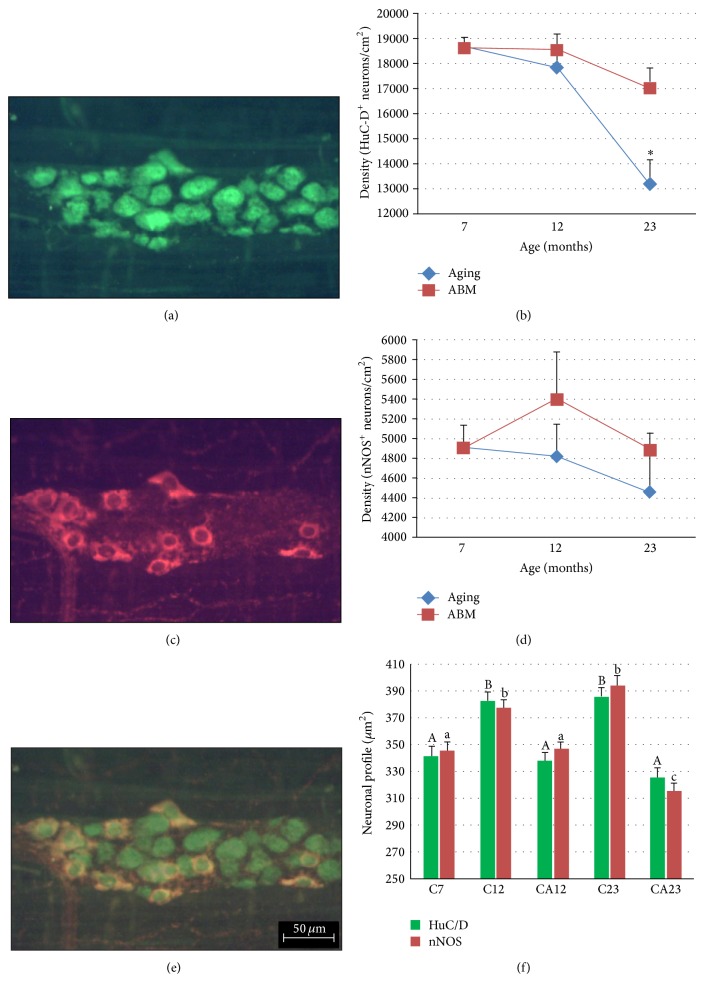
Myenteric ganglion of the jejunum in rats at 7 months of age: (a) HuC/D^+^ neurons and (c) nNOS^+^ neurons. (e) Overlay of images from (a) and (c). Density of (b) HuC/D ^+^ and (d) nNOS^+^ myenteric neurons. Neuronal profile (*μ*m²) of HuC/D^+^ (green) and nNOS^+^ (red) neurons shown in (f). Different letters in same-colored columns differ statistically (f). ^*^ *P* < 0.05, compared with C7 group. AGING, C7, C12, and C23 groups; ABM, C7, CA12, and CA23 groups. The results are expressed as mean ± standard error. Scale bar = 50 *μ*m.

Morphoquantitative changes were detected in HuC/HuD^+^ myenteric neurons as a result of aging. Significant reductions of neuronal density and the number of neurons/ganglia (29.2% and 32.4%, resp.) were observed in the C23 group compared with the C7 group. Supplementation with the aqueous extract of ABM had a positive effect on HuC/HuD^+^ myenteric neurons, in which the detected losses of density (8.5%) and number of neurons/ganglia (17%) in the C7 and CA23 groups were lower than in the 23-month-old control group. An increase in neuronal area (*P* ≤ 0.05) was observed in the C12 and C23 groups. The stable neuronal area in supplemented animals (CA12 and CA23 groups) compared with the C7 group indicates a neuroprotective effect of the ABM aqueous extract (Figure 3, Table 5).

The density of the subpopulation of nitrergic neurons (nNOS^+^) was preserved, regardless of age or supplementation. However, the neuronal profile of that population increased (*P* ≤ 0.05) with regard to age, with a positive effect of supplementation with the ABM aqueous extract at 12 months of age, with the exception of the CA23 group, in which the nitrergic neuron profile exhibited a significant reduction compared with the C7 and C23 groups (Figure 3).

**Figure 4:**
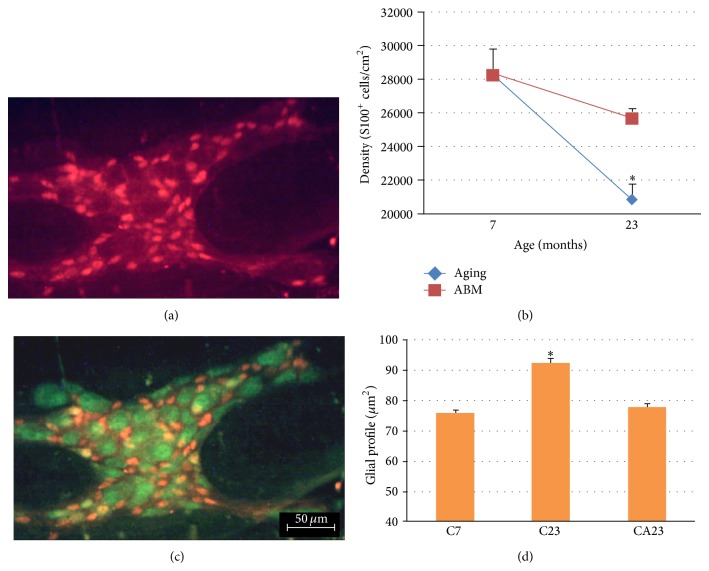
Myenteric ganglion of the jejunum in rats at 7 months of age: (a) S100^+^ glial cell body and (b) density of S100^+^ glial cells. HuC/HuD^+^ and glial S100^+^ glial neuron cell bodies (c) and glial profile presented in (d). ^*^ *P* < 0.05, compared with C7 and CA23 groups. AGING, C7, and C23 groups; ABM, C7, and CA23 groups. The results are expressed as mean ± standard error. Scale bar = 50 *μ*m.

The behavior of glial cells when comparing animals at 7 and 23 months of age revealed significant reductions of the density and number of glia of 26.3% and 27.3%, respectively, and an increase in the glial cell profile (*P* ≤ 0.05). A positive effect of the ABM aqueous extract was observed, with a 9% reduction of glial density, 14% reduction of the number of glia, and maintenance of a similar glial profile as the 7-month-old animals (Figure 4, Table 5). The glia/neuron ratio was maintained among the analyzed groups (Table 5).

## 4. Discussion

### 4.1. Biometric Parameters

The concentration of the ABM aqueous extract lyophilized used for animal supplementation during the aging process was 26 mg/animal/day (52 mg/kg). To standardize the supplementation dose, the commercial dosages ingested by humans were adopted as a reference, thus establishing a rate of intake of 4 g of dried mushrooms for a person who weighs approximately 60 kg. Additionally, the concentration corresponds to one-quarter of the dosage of mushroom extract used in acute treatments (200 mg/kg) described for rats [23].

Throughout the experimental period, a reduction of food intake (*P* < 0.05) was observed beginning at 12 months of age compared with the 7-month-old group, with maintenance of body weight and water intake. The reduced food intake in 12-month-old animals was 25%, whereas the reduction for 23-month-old animals was 21% compared with 7-month-old animals. Raul et al. [10] reported that food intake decreases by approximately 20% in rats during the aging process.

The association between final body weight and nasoanal length allows the calculation of the Lee index, a parameter comparable to body mass index. Together with the increase in retroperitoneal adipose tissue, the Lee index significantly increased in the C23 and CA23 groups compared with the C7 group, with no significant differences in periepididymal fat or total fat. The aging process in rats is associated with an increase in body fat [24] and decrease in muscle mass [25]. These changes mainly occur because of a decrease in basal metabolic rate and diminished physical activity [26, 27]. We detected marked hypoactivity in the animals throughout the experiment and a significant reduction of food intake, which explain the stable body weight.

Age, regardless of supplementation, led to an increase in small intestine length in the C23 and CA23 groups. This increase may cause slower intestinal transit, with the possibility of constipation, a frequently reported symptom among the elderly [7]. Similar results were reported by Phillips et al. [28] and Johnson et al. [29], who observed a progressive increase in small intestine length at 24 months of age in Fischer 344 and Sprague Dawley rats, respectively. In Wistar rats, maintenance [10] and a reduction [8] of the size of the small intestine related to aging were found in 29- and 15-month-old animals, respectively.

Prolonged supplementation with the aqueous extract of ABM did not influence the analyzed biometric parameters, indicating that prolonged intake was responsible neither for the onset of being overweight or obesity nor for any stimulus toward greater feed or water intake. Notably, the* Agaricus blazei* mushroom is regarded as a highly nutritious food because of its carbohydrate, protein, and fiber content and low levels of fat [30].

### 4.2. Biochemical Analysis of Blood Components

Aging significantly increased (*P* < 0.05) total plasma cholesterol levels in 23-month-old animals. This has been reported both for rats [31] and humans [32] in old age and is related to a higher incidence of diseases such as atherosclerosis [33].

One hypothesis for aging-related hypercholesterolemia involves the natural deficiency of growth hormone (GH) with aging. Parini et al. [31] reported a reduction of cholesterol in 18-month-old rats treated with GH, which indicates the pleiotropic effects of this hormone on lipoprotein metabolism.

Considering that plasma low-density lipoprotein levels are determined by the balance between its synthesis and elimination, some authors have suggested an increase in the intestinal absorption of cholesterol with aging [34]. However, Gälman et al. [35] found that aging-related hypercholesterolemia in rats was attributable to reduced excretion and not to the higher intestinal absorption of cholesterol.

Supplementation with the ABM extract showed a marked tendency to reduce total cholesterol in 23-month-old animals, but this result was not statistically significant. The beneficial effects of an ethanolic extract of the* Pleurotus ostreatus* mushroom on cholesterol metabolism were observed in rats supplemented by gavage at a dose of 300 mg/kg/day [36]. A similar effect was observed with supplementation with 200 mg/kg of* Agaricus bisporus* in rats for 3 weeks [37]. In humans supplemented with* Agaricus blazei *for 3 months using a hot aqueous extract at a daily dose of 3 g, Liu et al. [38] also reported a reduction of total cholesterol.

These results suggest a beneficial effect of mushrooms in reducing total cholesterol, but the form of extraction and daily dosage should be considered. We used a cold aqueous extract because it is safer with regard to the release of hepatotoxic substances previously described for extracts over 60°C [39]. We also supplemented animals with a low dosage administered over a prolonged period of time as a preventive measure and not as a treatment.

Aging also increased the serum levels of total proteins and globulins, but these levels are within the normal range for Wistar rats [40], with no influence of supplementation on these parameters. No effect of age or* A. blazei* supplementation on the blood levels of albumin or triglycerides was found.

Although glycemic levels are constant during the aging process, they were high (*P* < 0.05) in the CA23 group. The literature diverges with regard to glycemia in rodents, but we can discard the possible occurrence of a diabetic state because our values were less than 300 mg/dL, the threshold value that indicates diabetes in Wistar rats [41].

Aging did not alter the plasma levels of AST and ALT, indicating that aging did not lead to liver damage as described for rats [42] and humans [43]. Moreover, prolonged supplementation with the ABM aqueous extract did not influence these enzymes, which demonstrates the reliability of the concentration administered daily to the animals over a prolonged period of time.

Lee et al. [44] supplemented Fischer 344 rats for 2 years with* A. blazei* aqueous extract and observed no carcinogenic effects in several organs, such as the liver, brain, lungs, and intestine. Antimutagenic effects of the ABM aqueous extract were reported by Barbisan et al. [45] prior to the chemical induction of cancer in rats, demonstrating protection in the initial stage of liver carcinogenesis but no effect when administered in the postinduction period. Previous studies have also shown that* A. blazei* exerts a protective effect on liver function in rats following injury induced by carbon tetrachloride (CCl_4_; [46]).

We observed a reduction of the total plasma antioxidant capacity (TAC-ABTS) in 23-month-old animals. The reduction of this capacity in animals in advanced age is frequently reported in the literature [47]. When analyzing the effect of ABM supplementation on this parameter, we observed a tendency toward improved plasma antioxidant capacity in 12-month-old supplemented animals, but no significant differences were found between the C23 and CA23 groups.

### 4.3. Histological Analysis

The classic histological organization of the jejunum was maintained in rats in all of the groups [48, 49]. Nevertheless, morphometric alterations were detected in the aging process and with daily supplementation with the aqueous extract of* Agaricus blazei.* When analyzing the total intestinal wall, we observed a significant reduction of thickness in the 23-month-old animals; the same was observed for the mucosa tunic, villus height, and crypt depth in the intestine compared with 7-month-old rats (Table 3).

Reductions of the thickness of the intestinal wall and mucosa related to aging have been observed in rats [9, 10] and humans [50] as a consequence of the reduced food intake seen with aging, in which the availability of nutrients in the intestinal lumen demonstrably acts as a trophic factor [51].

According to Raul et al. [10], villus atrophy in Wistar rats between 12 and 29 months of age may be related to a decrease in the renewal rate of the epithelium, reflected by a corresponding reduction of crypt size. Höhn et al. [9] found villus atrophy of approximately 20–25% in 30-month-old rats, followed by architectural irregularity compared with 4-month-old rats.

Despite the reduction of these parameters, the cellular proliferation, reflected by the MetI, remained constant (Table 3). Equilibrium between cellular synthesis, migration, and extrusion processes leads to the maintenance of villus size and consequently the preservation of digestive and intestinal absorption capacity. Pluske et al. [52] highlighted that in situations of maintenance or reduction in the cell proliferation rate (MetI) associated with increased cell extrusion, in the apex of the villi, results in a reduction of their size. Our results indicate that absorption capacity was minimized and that supplementation with the ABM aqueous extract did not reverse this condition. The number of goblet cells per field was reduced (*P* < 0.05) with aging compared with the C7 group (Table 3), which is consistent with the reductions of villi and crypts discussed previously. Valenkevich and Zhukova [50] reported similar results for goblet cells in the duodenum in advanced-age humans. Caliciform cells are involved in the production of mucus that protects and lubricates the surface of the intestinal epithelium; therefore, reduced mucus secretion in response to aging [53] can damage the intestine by reducing the protective barrier against pathogens, facilitating their transport toward the inside of the mucosa and increasing the susceptibility to infection [51]. Fasting or dietary changes can also result in a reduction of the protective mucus layer [51].

Supplementation with the aqueous extract of* A. blazei* showed a tendency toward alleviating this condition in the CA23 group, but no significant differences were detected compared with the C23 group. This improvement may be explained by considering that the aqueous extract provided protein supplementation in the diet.

The muscular coat did not exhibit significant differences in thickness during the aging process (Figure 1). This result is consistent with Marese et al. [8], who attributed this result to a normal developmental condition imposed on the animal by diets with a normal protein content, regardless of age, although a reduction of intestinal motility associated with age was reported [53].

Supplementation with the ABM aqueous extract increased the muscular coat in old supplemented animals (CA23 group) compared with nonsupplemented animals (C23). The amount of proteins and amino acids present in the ABM aqueous extract [54, 55] likely contributed to the increase in the muscular coat during the long period of supplementation. Low-protein diets have been shown to reduce muscular coat thickness in Wistar rats [56].

### 4.4. Morphoquantitative Analysis of the Myenteric Plexus

We found that the density and number of cells per ganglion in the myenteric neuron (HuC/D^+^) and glial cell (S100^+^) population were significantly reduced at 7 and 23 months of age, whereas the density of the nitrergic subpopulation (nNOS^+^) remained stable.

Quantitative reductions of the enteric neuron population are frequently reported in the literature and may be linked to gastrointestinal problems, such as dysphagia, gastroesophageal reflux disorders, diarrhea, constipation, and fecal incontinence [57]. In addition to alterations in the ENS, damage to the sympathetic innervation of the plexi may be a possible mechanism for the decline in gastrointestinal motor function seen in old rats [58].

Reduction of the number of neurons has been observed in humans [59], guinea pigs [60], and rats [61, 62] in both the small intestine [8, 21, 28] and large intestine [21, 28]. The latter is seen as the most susceptible to aging-associated damage. In addition to effects in different species, organs, and segments, neuron populations and subpopulations can be affected differently.

Nitrergic neurons synthesize nitric oxide through nitric oxide synthase, and cholinergic neurons synthesize acetylcholine through choline acetyltransferase. These two neuronal subpopulations combined represent almost the entire myenteric neuron population in rats [63]. Phillips et al. [28] compared 3- and 24-month-old rats and found that neuronal loss in the small and large intestines occurred only with cholinergic neurons, thus corroborating the data obtained in the present study. Neurons marked by the pan neuronal marker anti-HuC/D in the cytoskeleton [64] were reduced by 29.2% in 23-month-old animals compared with the C7 group. The density of the nitrergic subpopulation was not significantly reduced, supporting the hypothesis that certain neuron classes may be more susceptible to aging than others [61, 65].

The cell profile analysis showed that the aging process significantly increased the cell body area for both HuC/HuD^+^ and nNOS^+^ neurons beginning at 12 months of age, although no quantitative alterations were detected during that period, which remained unchanged until 23 months of age. Age-related neuronal cell body hypertrophy was also described by Marese et al. [8] and Schoffen and Natali [14], who compared the duodenum in 3- and 14-month-old Wistar rats and the proximal colon in 3- and 12-month-old Wistar rats, respectively. This was considered a neuronal adaptation attributable to the reduced number of neurons.

Notably, despite maintaining their density, the changes in the cell profile of nitrergic neurons indicated that they were completely spared from the effects of aging. Phillips et al. [28] used the NADPH-diaphoresis technique and observed an increase in the cell body area of nitrergic neurons in the colon and rectum in 24-month-old Fischer 344 rats, with no alterations in the small intestine. These data differ from the results obtained in the present study.

The ENS changes that occur with aging may be related to a reduction of neurotrophic factors secreted by glial cells, which are important in neuronal development and maintenance [13]. In our work, glial loss was proportional to myenteric neuronal death. The glia:neuron ratio did not change over the different ages studied. The same pattern of cellular death was observed by Phillips et al. [21], who performed double-HuC/HuD-S100 immunostaining in Fischer 344 rats at 6 and 26 months of age, suggesting the interdependence of these two cell types.

The glial profile was also altered as a consequence of aging. An increase was observed in the mean cell body area in 23-month-old rats compared with 7-month-old rats. The progressive hypertrophy of astrocytes immunolabeled by the S-100 protein in the central nervous system also occurs frequently during the aging process in rats [66]. However, that population in the enteric nervous system has been seldom evaluated.

The quantitative reduction of cells present in enteric ganglia may be directly related to oxidative stress because of the higher production of free radicals and a decrease in the activity of antioxidant enzymes [1] that occurs during the aging process. Thrasivoulou et al. [12] performed* in vitro* analyses of myenteric neurons in Sprague-Dawley rats and found that the start of cellular death is linked to higher intraneuronal levels of reactive oxygen species (ROS).

Given that the total plasma antioxidant capacity (TAC) was reduced in 23-month-old rats and that the ABM aqueous extract has demonstrable antioxidant potential [55], we infer that the extract efficiently preserved neurons and glia in the myenteric plexus in old animals. No differences were detected in the number or profile of the cell bodies of glial cells and myenteric HuC/D neurons^+^ in the supplemented groups (CA12 and CA23) compared with the 7-month-old group.

Despite the numeric maintenance of nitrergic neurons during aging, atrophy was detected in the middle area of cell bodies in that neuronal subpopulation in 23-month-old supplemented animals compared with the 7-month-old group.

The possible involvement of specific components of the ABM aqueous extract, such as polyphenols, might justify this reduction because of their reported ability to sequester nitric oxide* in vitro* [67] and* in vivo* [68]. van Acker et al. [67] reported the neuroprotective effects of polyphenols. Epigallocatechin, a polyphenol isolated from green tea administered intraperitoneally in rats, attenuated oxidative stress by reducing the expression of nNOS and NADPH-d in parasympathetic ganglion neurons that extrinsically innervate the digestive tube in rats following hypoxia.

The precise mechanism by which* A. blazei* prevents neuronal death is still unclear. Nevertheless, considering that neuronal death can be a consequence of glial death [69], we can infer that the functions of glial cells were preserved by prolonged intake of the ABM aqueous extract during the aging process.

One hypothesis is that antioxidant compounds contained in the ABM aqueous extract have direct actions on free radicals generated in enteric glial cells. Given that they are similar to central nervous system (CNS) astrocytes with regard to their morphological and immunohistochemical characteristics [70, 71], this hypothesis can be supported by the results obtained by Sharma et al. [72], which indicate a positive effect of flavonoids on astrocytes through the modulation of glial fibrillary acidic protein (GFAP) and glutamine synthetase, with involvement in protective events, reducing apoptosis in a neuronal culture. Another hypothesis is that the chronic intake of glutamic acid (or glutamate) found in the aqueous extract of* A. blazei* has indirect antioxidant effects [54, 73]. Glutamic acid can be converted into glutamine, a precursor of glutathione, one of the most powerful cellular antioxidants. This substance is produced and released by enteric glial cells for neuronal preservation in cases of oxidative stress [74]. Moreover, Muyderman et al. [75] demonstrated that mitochondrial glutathione is essential for preserving the viability of astrocytes in the SNC during conditions of increased levels of free radicals, which supports our hypothesis.

A positive relationship between antioxidant compound supplementation and enteric neuroprotection in diabetic neuropathy has been reported by several authors. This diabetic condition, similar to aging, is associated with oxidative stress as one of its main degenerative factors [3]. Among the supplements studied in experimental models of diabetic neuropathy are ascorbic acid [76], *α*-tocopherol [77], L-glutamine [78],* Ginkgo biloba* [79], and quercetin [80].

In aging, a neuroprotective effect was detected in the ENS in the small intestine [81] and large intestine [82] in rats supplemented with* Ginkgo biloba* extract. Similarly, [83] found that supplementation with ascorbic acid had a neurotrophic effect on myenteric neurons in old rats, suggesting neuroprotection.

## 5. Conclusions

Aging modifies biometric, blood, and morphofunctional parameters in the jejunum and causes morphoquantitative changes in the enteric nervous system. Prolonged supplementation with the aqueous extract of* Agaricus blazei *efficiently maintained myenteric plexus homeostasis, which positively influenced the physiology and prevented the death of the neurons and glial cells.

## Figures and Tables

**Table 1:** Characteristics of primary and secondary antibodies used for immunohistochemical analysis.

Primary antibody	Supplier	Dilution	Secondary antibody^*^
Anti-HuC/HuD (mouse)	Invitrogen, USA	1 : 500	Alexa fluor 488 (anti-mouse)
Anti-nNOS (rabbit)	Santa Cruz Biotechnology, USA	1 : 500	Alexa fluor 546 (anti-rabbit)
Anti-S-100 (rabbit)	Sigma, USA	1 : 200	Alexa fluor 546 (anti-rabbit)

^*^The secondary antibodies were utilized with 1 : 500 dilution and supplied by Invitrogen, USA.

**Table 2:** Body weight (BW), food intake (FI), water intake (WI), Lee index (ILee), weight of periepididymal (PER) and retroperitoneal (RET) fat, total fat (Σ), and small intestine length (SIL) in aging rats (C7, C12, and C23 groups) and aging rats supplemented with the aqueous extract of *A. blazei *(CA12 and CA23 groups). The results are expressed as mean ± standard error.

	C7	C12	CA12	C23	CA23
BW (g)	489.5 ± 8.72a^*^	492.9 ± 4.32a	483.7 ± 8.97a	510.3 ± 7.67a	513.5 ± 3.66a
FI (g)	28.26 ± 0.38a	21.34 ± 0.38b	21.92 ± 0.61b	22.66 ± 0.47b	24.06 ± 0.60b
WI (mL)	43.7 ± 1.16a	40.11 ± 2.00a	43.0 ± 4.19a	48.45 ± 1.55a	39.0 ± 2.17a
ILee	295.6 ± 2.72a	301.7 ± 3.83ab	292.5 ± 1.47a	307.3 ± 1.55b	308 ± 0.73b
PER (g/100 g)	1.54 ± 0.18a	1.84 ± 0.04a	1.66 ± 0.19a	1.72 ± 0.15a	1.67 ± 0.11a
RET (g/100 g)	1.56 ± 0.15a	1.98 ± 0.17a	1.61 ± 0.24a	2.54 ± 0.15b	2.26 ± 0.16ab
Σ (g/100 g)	3.10 ± 0.32a	3.82 ± 0.15a	3.28 ± 0.36a	4.26 ± 0.30a	3.94 ± 0.24a
SIL (cm)	112.8 ± 5.66a	103.2 ± 4.81a	97.0 ± 5.03a	125.8 ± 5.38b	113.5 ± 1.83ab

^*^Different letters in the same line indicate significant statistical difference (*P* < 0.05).

**Table 3:** Total cholesterol (TC), total protein (TP), globulin (GB), albumin (AL), triglycerides (TG), and glycemia (GL) in aging rats (C7, C12, and C23 groups) and aging rats supplemented with the aqueous extract of *A. blazei* (CA12 and CA23 groups). The results are expressed as mean ± standard error.

	C7	C12	CA12	C23	CA23
TC (mg/dL)	101.6 ± 10.51a^*^	109.7 ± 8.22ab	125.1 ± 13.37ab	166 ± 22.14b	140.3 ± 17.08ab
TP (g/dL)	6.04 ± 0.15a	7.03 ± 0.14b	6.7 ± 0.18b	6.9 ± 0.10b	6.85 ± 0.09b
GB (g/dL)	3.78 ± 0.06a	4.59 ± 0.11b	4.43 ± 0.17b	4.55 ± 0.08b	4.42 ± 0.14b
AL (g/dL)	2.27 ± 0.16a	2.43 ± 0.08a	2.27 ± 0.09a	2.35 ± 0.07a	2.42 ± 0.04a
TG (mg/dL)	135.4 ± 11.99a	147.4 ± 15.79a	146.2 ± 15.2a	166 ± 33.09a	145 ± 34.85a
GL (mg/dL)	124.5 ± 2.95a	111.8 ± 6.56a	120.1 ± 4.06a	132.3 ± 7.16a	150.8 ± 6.8b

^*^Different letters in the same line indicate significant statistical difference (*P* < 0.05).

**Table 4:** Intestinal morphometry: total wall (TW), mucosa (MC), and muscle (MM) tunicae, villus height (VH), crypt depth (CD), metaphase index (MetI), and number of goblet cells (GLC) in the jejunum in aging rats (C7, C12, and C23 groups) and aging rats supplemented with the aqueous extract of *A. blazei* (CA12 and CA23 groups). The results are expressed as mean ± standard error.

	C7	C12	CA12	C23	CA23
TW (*µ*m)	835.13 ± 1.97a^*^	760.65 ± 2.32b	805.95 ± 3.11c	802.91 ± 6.52c	806.29 ± 2.86c
MC (*µ*m)	700.85 ± 2.20a	639.61 ± 2.24b	674.99 ± 3.24c	648.92 ± 5.56b	641.19 ± 2.19b
MM (*µ*m)	100.8 ± 0.59a	101.1 ± 0.89a	98.61 ± 0.67a	103.2 ± 0.82a	113.9 ± 1.13b
VH (*µ*m)	494.03 ± 1.75a	449.10 ± 2.52b	464.42 ± 2.36c	458.05 ± 3.23c	420.02 ± 2.91d
CD (*µ*m)	274.20 ± 1.72a	218.61 ± 1.15b	212.31 ± 1.14c	244.00 ± 1.56d	235.50 ± 1.36e
MetI (%)	7.59 ± 0.35a	6.78 ± 0.57a	6.74 ± 0.34a	7.42 ± 0.52a	7.53 ± 0.32a
GLC^#^	72.09 ± 2.49a	75.34 ± 2.99a	74.48 ± 2.59a	53.07 ± 1.88b	60.98 ± 1.86b

^*^Different letters in the same line indicate significant statistical difference (*P* < 0.05). ^#^Values expressed as goblet cells per field.

**Table 5:** Number of neurons (HuC/HuD^+^) and glial cells (S100^+^) per ganglion and glia/neuron ratio in rats at 7 months of age (C7 group) and 23 months of age (C23 group) and rats supplemented with the aqueous extract of *A. blazei* (CA23 group). The results are expressed as mean ± standard error.

	C7	C23	CA23
HuC/HuD^+^/ganglion neurons	24.84 ± 0.74a^*^	16.77 ± 0.50b	20.44 ± 0.66c
S100^+^/ganglion glias	34.17 ± 0.98a	24.81 ± 0.67b	29.37 ± 0.90c
glias/neurons proportion	1.37 ± 0.03a	1.50 ± 0.12a	1.44 ± 0.07a

^*^Different letters in same line indicate significant statistical difference (*P* < 0.05).
